# Neural correlates of abnormal cognitive conflict resolution in major depression: An event-related potential study

**DOI:** 10.3389/fpsyt.2022.989924

**Published:** 2022-09-06

**Authors:** Ru-hong Sun, Jia-zhao Zhang, Sha-yu Jin, Chen-guang Jiang, Xue-zheng Gao, Jun Wang, Zhen-he Zhou

**Affiliations:** ^1^Department of Psychiatry, The Affiliated Wuxi Mental Health Center of Nanjing Medical University, Wuxi, Jiangsu, China; ^2^3 Grade 2019 Class 6, Basic Medicine College of Jinzhou Medical University, Jinzhou, Liaoning, China

**Keywords:** cognitive conflict resolution, major depression, lateralized readiness potential, P300, simon task

## Abstract

Abnormal cognitive conflict resolution has been considered as a critical element of executive dysfunctions inpatient with major depression (MD). Further clarifying whether there was a deficit at perceptual encoding stage or the early response-execution stage in conflict control function by event-related potential (ERP) technique in MD would be helpful in understanding the neural mechanism of MD. Participants included twenty-six depressed patients and twenty-six healthy controls (HCs). All participants measured with Hamilton Depression Scale (17-item edition, HAMD) and a Simon task. Electroencephalograms were synchronously recorded when performing the Simon task. The method of residue iteration decomposition was used to analyze the lateralized readiness potential (LRP) and P300 components, which contributed to divides ERP components into a stimulus-locked component (S-cluster), a response-locked component (R-cluster) and an intermediate component cluster (C-cluster) by using latency variability and time markers. Results showed that reactive times (RTs) for both groups were fastest in congruent trials, and slowest in incongruent trials; however, there is no difference in RTs under the three conditions between two groups. Accuracy Rate (ACC) for both groups were the highest in neutral trials, and the lowest in incongruent trials; ACC in MD group were all lower than that of HC group under three conditions. ERP data analyses showed that depressed patients had a deficit in activating the correct response, as reflected by reduced amplitudes of R-LRP, but no abnormality in LRP-S and P300-C. In conclusion, patients with MD present conflict control dysfunction (i.e., abnormal cognitive conflict resolution) at the early response-execution stage, not at perceptual encoding stage, which may be reflected by the reduced R-LRP amplitudes. The abnormal cognitive conflict resolution in activating the correct response might constitute an interesting treatment target.

## Introduction

Major depression (MD) is an incapacitating health care problem worldwide according to the illness-induced disability, and characterized by lowered mood, loss of interest and happiness, as well as impaired cognitive functions (1, 2). Of above typical symptoms, cognitive dysfunctions are the main determinant of patients’ health outcome in MD. A recent study had reported that 94 percent of depressed patients had cognitive dysfunctions during the onset of depression, and in remission of depression, they still had cognitive impairments (3). In clinical practice, as reduction of depressive symptoms, remediation of cognitive impairments also plays an important role in improving the prognosis of patients with MD (4).

Cognitive dysfunctions in MD includes several areas, the most common being mild to moderate deficiencies in attention, memory, learning, processing speed and executive function (5). Executive function, the complex cognitive control mechanism that coordinates the operation of various seed processes, dynamically regulates human cognition and behavior, and is essential for accomplishing most tasks in daily life. Previous studies indicated that when poor executive function persists in the remission phase of depression, it is an important residual symptom that affects an individual’s recovery (6, 7). Therefore, executive dysfunction is considered as a core target of new treatment strategies for MD (8, 9). Many studies have certified that conflict control, i.e., cognitive conflict resolution, is a critical element of executive function (10). Cognitive conflict resolutions include the information processing stage and the response selection stage (11, 12). Determining at which stage the impairment of conflict control functions in MD occurs may help clinicians to better understand the role of neurocognitive dysfunction in daily life and to target interventions. Previous researches showed that patients with MD present abnormal cognitive conflict resolution, for example, a study involved reactive time (RT) data of two-choice visual RT tasks showed that patients with MD does not affect the stimulus preprocessing stage, but affects the motor regulation stage (13). Another study which used Stroop task supported the opinion that late onset depressed patients are cognitively impaired and that this impairment persists in the period of early remission, namely, the impaired information processing occurs at an earlier, pre-response related stage (14).

Many neuropsychological tests, such as Simon task, Stroop task, Eriksen flanker task, have been used to measure the degree of cognitive conflict resolution (15–18). Although Simon, Stroop, and Eriksen flanker effect involve unrelated stimulus information and trigger response conflicts in some way, it has been proposed that Simon task is the only task that can completely control the manipulated relationship and possible conflict between cognitive representations, and it is the preferred tool to study the interaction between perception and action, as well as the research related to this interaction (19). Simon task may give a better answer to whether the conflict control of depression occurs in the information processing stage or the response selection stage. In Simon task, subjects were asked to respond left- or right-hand according to the color of the stimulus appearing to the left or right of the central fixation (20). Although the stimulus position is irrelevant to the task, the RT to the ipsilateral stimulus (stimulus on the left, reaction hand on the left) is shorter than the contra lateral stimulus (stimulus on the left, response hand on the right), and accuracy is higher. A recent study investigated the cognitive and somatic symptoms of premenstrual dysphoric disorder (PMDD) in the early luteal and later luteal phase, and results showed that people with PMDD had a poor performance in Simon’s task (i.e., executive function was impaired) during the later luteal phase, and their cognitive reappraisal positively correlated with executive function and negatively associated with depression (21).

Event-related potential (ERP) is a useful tool for studying motion preparation processes because of its high temporal precision, down to the “millisecond” level, compared to other imaging techniques such as functional Magnetic Resonance Imaging (fMRI)and Near Infrared Spectroscopy (22). Lateralized readiness potential (LRP), which recorded from scalp electrode site C3 and C4, is a movement-related brain evoked potential that reflects hand-specific motor preparation in the pre-central motor cortex, and it has been used to evaluate the Simon effect (23). A study indicated that a negative potential appeared in the contra lateral scalp of the reactive hand before the movement of the hand, and its maximum amplitude was located in the center of the scalp corresponding to the position of the primary motor cortex (24). Another study showed that when the subjects knew whether they needed to make a “left” or “right” response, the brain activity showed asymmetry, which reflected the subjects’ preparation for specific actions (25). Previous studies have found that when the position of the stimulus and responder hand is inconsistent, the RT is longer, and a positive potential deflection named Gratton-dip, can be observed in the early stages of LRP, indicating that the wrong response is activated (26), because the ipsilateral response tendency must be inhibited first, which is a competitive process that produces a conflict of reactions. When the stimulus and the responding hand are in the same position, the RT is short and the correct response is directly activated.

The time from the presentation of the stimulus to the beginning of the LRP (stimulus locking LRP, S-LRP) provides a measure of the time spent in perceptual and cognitive processes that take place before selecting a response. The time interval between the start of LRP and the dominant reaction (reaction locking LRP, R-LRP) provides an indicator of reaction execution time (27). The information provided by LRP is helpful to determine whether the interference effect occurs in the perceptual stage or the response selection and execution stage of processing (19). Therefore, LRP can be used as an index to study the influence of psychological processing. Thomas et al. confirmed that pergolide, a dopamine agonist, could effectively shorten the latency of S-LRP, but had no effect on the latency of R-LRP, indicating that this drug could improve the speed of information processing, but had no effect on the speed of execution (28). Houlinhan et al. proved that smoking (nicotine) can shorten the latency of both S-LRP and R-LRP, and increase maximum amplitudes (29). In addition, Roggeveen et al. also applied LRP to study that response slowing in the elderly is mainly due to slowed preparation and execution of responses (R-LRP) rather than cognitive processing (S-LRP) (30). Above studies conclude that the latencies and amplitudes of S-LRP and R-LRP are affected by age, drugs and other factors. It is worth noting that the overlap of LRP related to visual motor priming and response selection-related LRP affects the onsets of correct LRP activation. Stürmer et al. found that the residue iteration decomposition (RIDE) was a useful method to separate them (31). RIDE divides ERP components into a stimulus-locked component (S-cluster), a response-locked component (R-cluster) and an intermediate component cluster (C-cluster) by using latency variability and time markers (32, 33).

The theory of event coding details how perception and action are associated (bound) in event file, which can be separated from pure stimulus and response-related processes in Electroencephalogram (EEG) signals (34–36). In other words, this translation process can be detected in C-cluster signal, but not in S-cluster and R-cluster data (37–39). Compared to the undecomposed EEG, Kleimaker et al. found that event file binding related modulations were evident in the C-cluster in the ERP P300 component (40). P300 is the event-related potential evoked by task-related stimuli, which is a positive waveform with an extreme value located in the parietal lobe. In contrast to studies considering the P300 as a stimulus evaluation index (41), other studies emphasized that the P300 is sensitive to the duration of the response selection phase (42, 43), however, subsequent studies have shown that P300 reflect a link between stimulus evaluation and responding processes (44, 45). Specifically, P300 reflect reactivation of well-established S–R links (46, 47). Several studies have found that patients with depression have a decrease in P300 (48–50). Thus, in event files, the modulation of the P300 in the C-cluster seems to be the most important neurophysiological marker (40).

Up to date, no studies on LRP characteristics of the translation process between perception and response in MD have been reported. Additionally, whether there was a deficit at perceptual encoding stage or the early response-execution stage in conflict control function in MD remained unclear. Further investigating the LRP characteristics of the translation process between perception and response in MD would be helpful in understanding the neural process of the Simon effect. Furthermore, it has implications for understanding the aetiology and the critical treatment targets in MD.

In present study, the participants included depressed patients and healthy controls (HCs), and LRPs were used to investigate the neural process of the Simon effect. We used S-LRP to represent the perceptual coding stage, the P300 C-cluster to represent the transformation stage of stimulus and response, and R-LRP to represent the response execution stage, which may be reflected by the differences in the amplitude and latency of these EEG components. The purpose of the current study was to explore the neural mechanism of the cognitive processing of the abnormal cognitive conflict resolution in MD.

## Materials and methods

### Time and setting

The present study was implemented from May 01, 2020, to December 31, 2021, in the Department of Psychology, Affiliated Wuxi Mental Health Center of Nanjing Medical University, Wuxi City, Jiangsu Province, the People Republic of China. The study was approved by the Ethics Review Committee of Affiliated Wuxi Mental Health Center of Nanjing Medical University and was in accordance with the Helsinki Declaration.

### Diagnostic approaches and participants

Participants consisted of depressed patients and HCs. The Structured Clinical Interview for DSM-IV (SCID, Chinese version) is a reliable psychiatric diagnosis for adults according to the Diagnostic and Statistical Manual, Fourth Edition (DSM-IV). All our patients and healthy controls were assessed by two senior clinicians who were present at the same time and did not dispute the assessment results. The Hamilton Rating Scale for Depression (51) is a 17-item clinician-rated tool that measures the severity of depression in patients and is one of the most widely used outcome measures in depression (52). The Cronbach’s alpha coefficient of the HAMD scale in this experiment was 0.86, with good internal consistency. The criteria for the MD group were as follows: (a) only meet the criteria of DSM- IV) for MD; (b) age from 18 years old to 55 years old; (c) HAMD (17-item edition) scores ≥ 17; (d) had not taken medication which damaged cognitive function, such as atropine, benzodiazepine etc., for the last two weeks; (e) had no electroconvulsive therapy or modified electroconvulsive therapy within the last month; (f) had normal vision; (g) had no diagnosis of alcohol, nicotine, or other substance dependency, any kind of neurological disorder that might have an effect on the central nervous system. The criteria for the HC group were as follows: (a) did not meet the criteria of any DSM-IV axis I disorder or personality disorders according to the Structured Clinical Interview for DSM-IV (SCID-4, Chinese version); (b) age from 18 years old to 55 years old; (c) HAMD (17-item edition) scores ≤ 7; (d) had no diagnosis of alcohol, nicotine, or other substance dependence; (e) had normal vision; and (f) had no diagnosis of any kind of any kind of neurological disorder that might have an effect on the central nervous system. All participants provided written informed consent to participate in this study. For depressed patients, whose capacity to consent was compromised, researchers obtained consent from their next of kin or guardians. Each participant was compensated 43.09 United States Dollars.

### Experimental procedure

**FIGURE 1:**
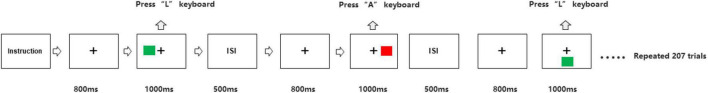
Experimental procedure of Simon task. The green or red square was presented on right, left or below side of the fixation on the computer screen. For the green square, participants had to press the “L” keyboard with right hand. For red square, participants had to press the “A” keyboard with left hand.ISI, inter-stimulus interval.

The participants sat at a distance of 50 centimeter (cm) in front of the computer screen. We programmed Simon task paradigm through E-Prime 3.0 software (Psychology Software Tools Incorporated, Pittsburgh, United States). As shown in Figure 1, first, a central fixation displayed 800 millisecond (ms) in a black screen, then a red or a green square appeared in one of the left, right or bottom positions to the fixation for 1,000 ms. Participants were asked to press the “A” keyboard key as quickly and accurately as possible corresponding to the red square was presented, and the “L” key when the green square was presented. All squares were Go stimuli. After the stimulus, the screen remained blank for 500 ms, and then the next circulation was presented. In the congruent trials, stimulus localization and correct response localization were on the same side. In the incongruent trials, they were on opposite sides, while in the neutral trials, the lower position of the central stimulus was not associated with any response side. There were therefore three different conditions mixed in a block with equal presentation probabilities, congruent stimuli, neutral stimuli, and incongruent stimuli. Participants performed three blocks of 70 stimulus presentations each. In both groups, behavioral data, including accuracy and RTs were extracted.

### Electroencephalogram recording and analysis

EEG recordings were taken from 64 Ag/AgCl scalp electrodes placed in international 10–20 system, using a BrainCap (EasyCap, Herrsching, Germany) with online band-pass-filtered from 0.05 to 100 Hertz (Hz). Vertical and horizontal electrooculography were recorded at three electrodes located below the left eye and at the lateral canthus of both eyes. The EEG signal recorded at the of 500 Hz sample rate, using the central reference of the forehead and the average value of the left and right mastoid as the re-reference. And the ground electrodes were placed under the left clavicle site. EEGs were filtered off-line with a low-pass filter at 0.5–20 Hz.

Data were segmented by stimulus from –200 ms to 1,200 ms. For waveforms of P300 and LRP (in which 0 ms corresponds to the onset of stimulus presentation), a baseline correction was set from –200 ms to 0 ms and removed all segments with amplitudes below –80 μV or above 80 uV. ERPs were only derived from the correct response of the participant’s RT within 100–1,000 ms. We had a visual inspection of the data, removing channels with significant artifacts while monitoring and correcting eye movements using independent component analysis (ICA).

RIDE decomposes the ERPs into S- and R-clusters according to the latency information of the stimulus and response onsets (31, 53). The C-cluster’s latency information is estimated in every single-trial and iteratively improved. RIDE algorithm uses a time window function to extract the waveform of each LRP component, which should cover the existence range of the corresponding component. In the current study, the extracted time window was from 0 to 500 ms for the S-cluster, the C-cluster from 50 to 700 ms and for R component was –300 to 300 ms around the response. LRPs were derived for each component cluster according to the widely recognized formula: *[(C4 - C3) left hand* + *(C3 - C4) right hand]/2* (54).

The latency onset of stimulus-locked LRPs is as the point in time when the amplitude of the difference waveform reached 50 percent of the peak amplitude (55). We used a criterion 90 percent of the peak amplitude to measure the onset of response-locked LRPs. In order to reduce the influence of measurement errors and outliers, the jackknife-based procedure was used to measure the LRP onset, and *F* values should be corrected as follows: *Fc* = *F/(n - 1)^2^* (56). The mean amplitude was measured after the onset of the stimulus corresponding to the correct response activation, following time windows were applied for quantification: LRP-S: 140–280 ms for all conditions; LRP-R: the LRP-R component was quantified after the onset of the congruent stimulus 370–400 ms for congruent condition; 380–410 ms for incongruent condition.

**FIGURE 2:**
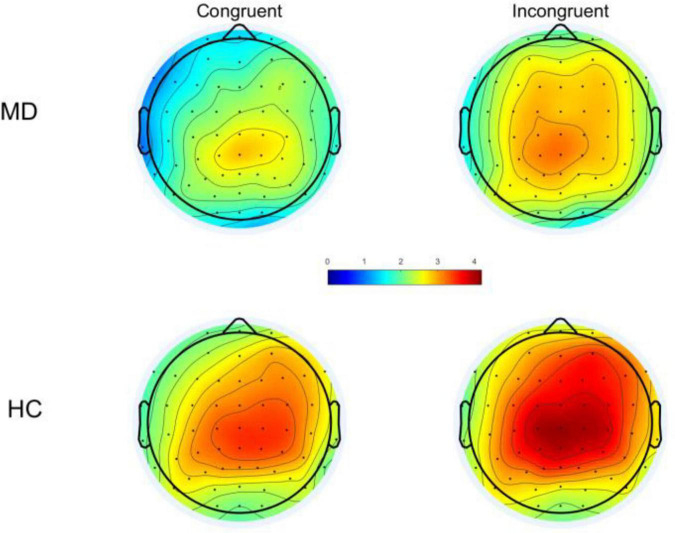
Following stimulus onset, topographical distribution of P300 within a time window of 200–500 ms under congruent condition and 250–550 ms for incongruent condition. MD, major depression; HC, healthy control.

Based on the topography (Figure 2), the maximum value can be seen at the Cz electrode, and combined with the reference (33), the P300 was measured at the Cz, and the time window of the P300 component determined by visual inspection: 200–500 ms for congruent condition; 250–550 ms for the incongruent condition; the wave amplitude was calculated as the average wave amplitude in the time interval.

### Statistical analysis

The IBM SPSS Statistic version 26 (IBM Corp., Armonk, NY, United States) was used for data analysis. Comparisons of mean age and education years were conducted between the patient group and the HC group with independent *t*-tests. Comparison of gender was conducted with the Pearson *chi-square* test. A repeated measures analysis of variance (ANOVA) was used to compare RTs, Accuracy Rate (ACC; percent of correct responses/hits) and ERP components (LRP-S, LRP-R and P300-C), with group as a between-subject factor and condition as a within-subject factor. Alpha values of 0.05 were considered significant and probability values were adjusted with the Greenhouse-Geisser epsilon correction for non-sphericity. Bonferroni correction was performed as *post hoc* analyses if needed. The Pearson’s correlation analysis was conducted between the behavioral data and amplitudes of LRP-S, P300-C and LRP-R under both conditions (congruent vs. incongruent). Alpha values of 0.05 were considered significant. Effect sizes were estimated using $\eta_{p}^{2}$.

## Results

### Analysis of demographic and clinical data

According to the inclusion and exclusion criteria, a total of twenty-six depressed patients (fourteen females) and twenty-six HCs (eleven females) completed the study and were analyzed. As shown in Table 1, there were no significant differences between MD group and HC group in mean age, education level, handedness, and sexual ratio. For depressed patients, the mean Fluoxetine-equivalent dose was (38.5 ± 1.3) mg/d as calculated according to the recent study (57).

**TABLE 1:** Demographic and clinical data [mean (SD)] for MD and HC group.

Variable	MD	HC	Statistic	*p*
Age (years)	36.65(9.90)	39.62 (4.20)	*t* = 1.396	0.172
Gender (M/F)	15/11	12/14	$\chi_{p}^{2}$ = 0.690	0.405
Education	12.69 (2.72)	13.12 (3.33)	*t* = 0.502	0.618
Handedness (R/M/L)	10/8/8	9/10/7	$\chi_{p}^{2}$ = 0.342	0.843
Duration of illness (years)	3.37(2.33)	–	–	–
HAMD-17	25.61(2.16)	–	–	–
Medicine (V/M/E/M/D/S/F)	7/1/3/2/4/4/5	–	–	–

MD, major depression group; HC, healthy control group; SD, standard deviation; R, right; M, mixed; L, left; HAMD-17, Hamilton Depression Scale (17-item edition). Medicine (V, Venlafaxine; M, Mianserin; E, Escitalopram; M, Mirtazapine; D, Duloxetine; S, Sertraline; F, Fluoxetine).

### Analysis of behavioral data

**FIGURE 3:**
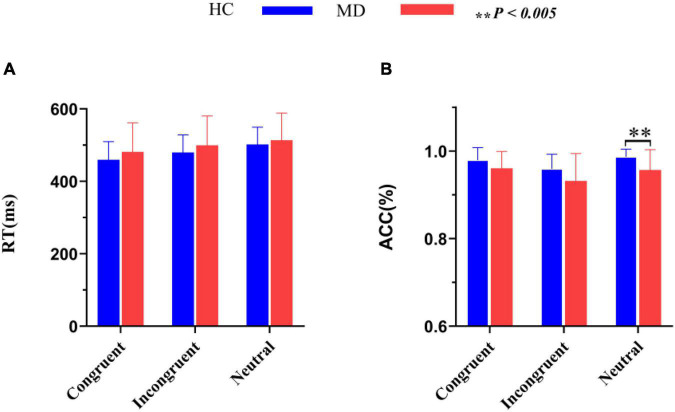
Comparisons of RT **(A)** and ACC **(B)** between the MD group and HC group. MD, major depression group; HC, healthy control group; RT, reaction time; ACC, accuracy rate (percent of correct responses/hits). ^**^P < 0.005.

RTs and ACC are shown in Figure 3 and Table 2. Using RTs as dependent variables, a 2-group (MD vs. HC) × 3-condition (Congruent vs. Neutral vs. Incongruent) repeated-measures ANOVA revealed no interaction of group × condition (*F_2,100_* = 1.42, *p* = 0.25, $\eta_{p}^{2}$ = 0.028), however, there was a significant “condition” main effect (*F_2, 100_* = 72.43, *p* = 0.001, $\eta_{p}^{2}$ = 0.592). RTs for both groups were fastest in congruent trials, and slowest in incongruent trials. Although depressed patients had slightly longer RTs than HCs in all three conditions, the “group” main effects were also not significant (*F_1, 50_* = 0.98, *p* = 0.16, $\eta_{p}^{2}$ = 0.019).

**TABLE 2:** Comparisons of RTs (ms) and ACC [mean (SD)] between the MD group and HC group.

Condition	RTs		ACC	
		
	MD	HC	MD	HC
Congruent	481.23 (80.39)	459.73 (49.91)	0.96 (0.029)	0.98 (0.029)
Incongruent	513.48 (75.10)	501.78 (47.57)	0.93 (0.062)	0.95 (0.033)
Neutral	499.47 (80.98)	479.87 (48.34)	0.96 (0.047)	0.99 (0.018)

MD, major depression group; HC, healthy control group; SD: standard deviation; RTs, reaction times; ACC, accuracy rate (percent of correct responses/hits).

Using accuracy as dependent variables, a 2-group (MD vs. HC) × 3-condition (Congruent,Neutral vs. Incongruent) repeated-measures ANOVA was conducted. It revealed a significant “condition” main effect (*F_2, 49_* = 9.91, *p* = 0.000, $\eta_{p}^{2}$ = 0.288) and a significant “group” main effect (*F_1, 50_* = 7.52, *p* = 0.008, $\eta_{p}^{2}$ = 1.31); The interaction for group × condition was not significant (*F_2, 49_* = 1.092, *p* = 0.344, $\eta_{p}^{2}$ = 0.043). *Post hoc* tests on the task revealed a significant higher ACC for the HC group than the MD group in the neutral condition (*F_1_,_50_* = 9.606, *p* = 0.003, $\eta_{p}^{2}$ = 0.161), but not in the congruent (*F_1_,_50_* = 3.927, *p* = 0.053, η*2* = 0.073) and incongruent condition (*F_1_,_50_* = 3.934, *p* = 0.053, $\eta_{p}^{2}$ = 0.073). ACC for both groups were the highest in neutral trials, and the lowest in incongruent trials. ACC in MD group were all lower than that of HC group under three conditions.

### Analysis of event-related potentials data

**FIGURE 4:**
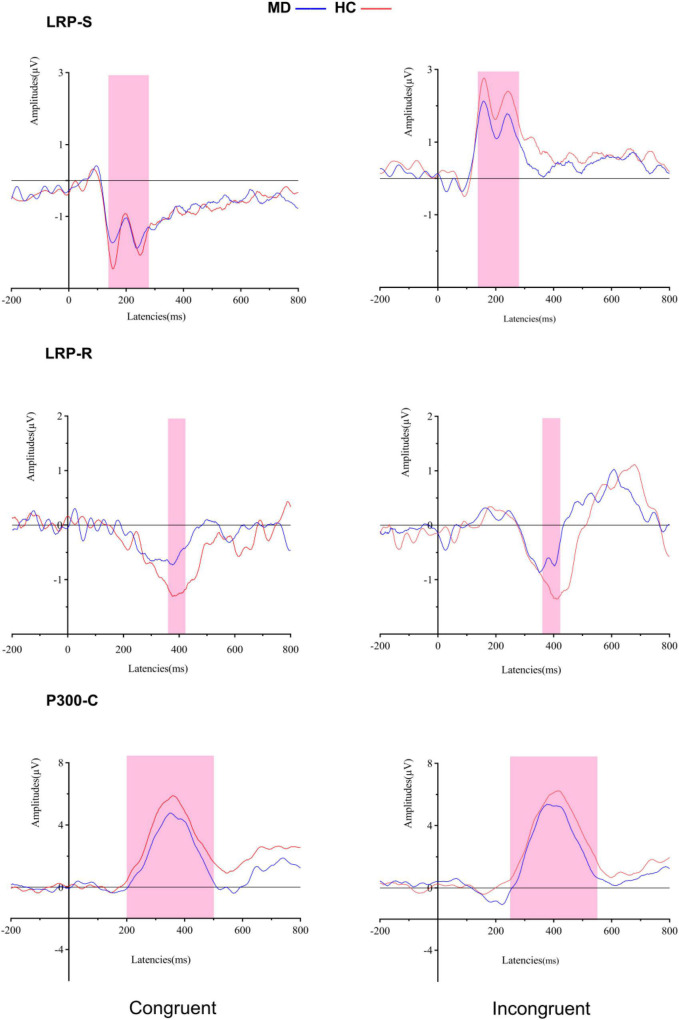
Grand averaged ERPs of both groups under congruent and incongruent condition (LRP-S is represented for the perceptual coding stage: 140–280 ms for all conditions; LRP-R is represented for the response execution stage: 370–400 ms for congruent condition, and 380–410 ms for incongruent condition). P300-C at the Cz electrode site is represented for transformation stage of stimulus and response (200–500 ms for congruent condition, and 250–550 ms for incongruent condition). MD, major depression group; HC, healthy control group.

As shown in Figure 4. Using LRP S-cluster, LRP R-cluster and P300 C-cluster amplitudes and latencies as dependent variables respectively, a 2-group (MD vs. HC) × 2-condition (Congruent vs. Incongruent) repeated-measures ANOVA was conducted.

#### Lateralized readiness potential S-cluster

For amplitudes, it revealed a significant “condition” main effect (*F_1, 50_* = 94.624, *p* = 0.000, $\eta_{p}^{2}$ = 0.654), but no significant “group” main effect (*F_1, 50_* = 1.621, *p* = 0.209, $\eta_{p}^{2}$ = 0.031); the interaction for group × condition was not significant (*F_1, 50_* = 1.034, *p* = 0.314, $\eta_{p}^{2}$ = 0.020). For onset latencies, it revealed no significant “condition” main effect (*F_1, 50_* = 281.54, *Fc_1, 50_* = 0.033, *p_*adjusted*_* > 0.05) and no significant “group” main effect (*F_1, 50_* = 68.33, *Fc_1, 50_* = 0.026, *p_*adjusted*_* > 0.05); the interaction for group × condition was not significant (*F_1, 50_* = 22.18, *Fc_1, 50_* = 0.009, *p_adjusted_* > 0.05).

#### Lateralized readiness potential R-cluster

For amplitudes, it revealed a significant “group” main effect (*F_1, 50_* = 8.405, *p* = 0.006, $\eta_{p}^{2}$ = 0.144) and no significant “condition” main effect (*F_1, 50_* = 0.364, *p* = 0.549, $\eta_{p}^{2}$ = 0.07); the interaction for group × condition was not significant (*F_1, 50_* = 0.212, *p* = 0.647, $\eta_{p}^{2}$ = 0.04). *Post hoc* tests showed that the amplitude of LRP-R in the MD group was significantly lower than the HC group in congruent (*F_1_,_50_* = 4.296, *p* = 0.043, η*2* = 0.079), and incongruent conditions (*F_1_,_50_* = 6.646, *p* = 0.013, $\eta_{p}^{2}$ = 0.117).

For onset latencies, it revealed no significant “condition” main effect (*F_1, 50_* = 2.502, *Fc_1, 50_* = 0.0009, *p_*adjusted*_* > 0.05) and no significant “group” main effect (*F_1, 50_* = 87.039, *Fc_1, 50_* = 0.033, *p_*adjusted*_* > 0.05); the interaction for group × condition was not significant (*F_1, 50_* = 1.365, *Fc_1, 50_* = 0.0005, *p_*adjusted*_* > 0.05).

#### P300 C-cluster

For amplitudes, it revealed no significant “group” main effect (*F_1, 50_* = 1.837, *p* = 0.181, $\eta_{p}^{2}$ = 0.035) and no significant “condition” main effect (*F_1, 50_* = 1.499, *p* = 0.227, $\eta_{p}^{2}$ = 0.029); the interaction for group × condition was not significant (*F_1, 50_* = 0.021, *p* = 0.884, $\eta_{p}^{2}$ = 0.000). For onset latencies, it revealed a significant “condition” main effect (*F_1, 50_* = 20.224, *p* = 0.000, $\eta_{p}^{2}$ = 0.288), but no significant “group” main effect (*F_1, 50_* = 0.16, *p* = 0.901, $\eta_{p}^{2}$ = 000); the interaction for group × condition was not significant (*F_1, 50_* = 0.796, *p* = 0.377, $\eta_{p}^{2}$ = 0.016).

### Analysis of correlations

**FIGURE 5:**
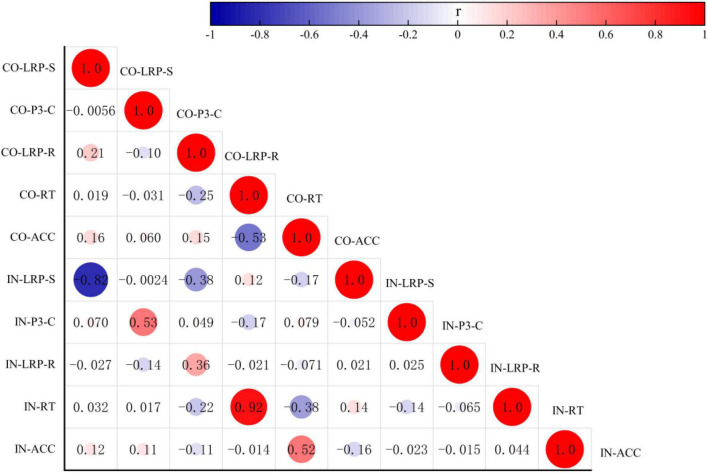
Analysis of correlation among the behavioral data RT, ACC, and the amplitude of the ERP components LRP-S, LRP-R, and P300-C under congruent and incongruent conditions. CO, congruent condition; IN, incongruent condition.

The Pearson correlation analysis method was used to analyze the correlations among the behavioral data, RTs, ACC, and the amplitudes of the ERP component LRP-S, LRP-R, and P300-C under congruent and incongruent conditions. There were no correlations between the behavior data (RTs vs. ACC) and the amplitudes of the ERP components (LRP-S, LRP-R, and P300-C) respectively. However, as shown in Figure 5, the RTs under the two conditions had a positive correlation (*r* = 0.924; *p* = 0.000), the ACC under the congruent condition was either significantly correlated with the RTs under congruent and incongruent condition (*r* = –0.526, –0.375; *p* = 0.000, 0.006) or significantly correlated with the ACC under incongruent (*r* = 0.522; *p* = 0.000). The amplitudes of ERP components (LRP-R and P300-C) had positive correlations under different conditions (*r* = 0.357, 0.528; *p* = 0.009, 0.000), the amplitudes of the ERP component (LRP-S) had a negative correlation under two conditions (*r* = –0.818; *p* = 0.000).

### Supplementary analyses

In order to support the unique role of the LRP-R in major depression, we conducted a logistic regression predicting group status utilizing LRP-S, P300, and LRP-R. Two separate models were conducted utilizing congruent elicited ERPs and incongruent ERPs to be more straightforward. Under congruent condition, using MD as dependent variables, the amplitudes of LRP-S, P300, and LRP-R as independent, all *p* value are more than 0.072; LRP-R alone as the independent variable, *p* value are more than 0.051. Under incongruent condition, using MD as dependent variables, the amplitudes of LRP-S, P300, and LRP-R as independent, the amplitudes of LRP-R were significant for predicting major depression (OR = 1.859, 95% CI 1.133–3.050, *p* = 0.014), alone using the amplitudes of LRP-R as independent were also significant (OR = 1.746, 95% CI 1.096–2.783, *p* = 0.019).

## Discussion

This study is first time to investigate the depressed patients’ neural process of the Simon effect, i.e., whether there was a deficit at perceptual encoding stage or the early response-execution stage in conflict control function, which was reflected by LRP characteristics of the translation process between perception and response. In addition, our study employed residue iteration decomposition, which is an important technique, to analyze the LRP component. Our study found that RTs for both depressed patients and HCs were fastest in congruent trials, and slowest in incongruent trials; however, there is no difference in RTs under the three conditions (Congruent, Neutral and Incongruent) between two groups. ACC for both groups were the highest in neutral trials, and the lowest in incongruent trials; ACC in MD group were all lower than that of HC group under three conditions.

Our behavioral outcome is consistent with previous research (15, 16, 58). It indicates that the Simon task is meaningful as a conflicting operation. Congruent trials contribute to convert spatial stimulus locations into motor responses (59, 60). Importantly, observing that differences when there is no response do not necessarily involve the underlying cognitive processes that occur in the brain during the performance of a task (61).

The advantage of LRP is that it can reveal the temporal characteristics of cognitive processing in the brain, which makes it easier to distinguish between the stages and time courses of mental processing. In this study, LRP S-cluster as stimulus-related measurement component did not differ between MD patients and HCs. It may have a very high contrast with our stimulus; however, it does not affect the experimental results due to the nature of the stimulus above the threshold. Therefore, there will be no difference in the ability to visually process between depressed patients and HCs. Specifically, the depressed patients’ attention distribution of the color and location of stimuli is the same as HCs. Our results showed that the depressed patients have no obvious dysfunction in transformation stage of stimulus and response, which were reflected by the P300 C-cluster amplitude. Existing studies show decreased γ-aminobutyric acid (GABA) levels in cerebrospinal fluid and plasma in depressed patients (62, 63). A recent study used fMRI showed that the concentration of GABA + /N-acetylaspartate in striatum and ACC did not seem to modulate event file binding effect, which provides evidence for our finding (64).

Our study displayed that there was a difference in the amplitude associated with response to stimuli between MD group and HC group incongruent conditions, which indicates a difference in the ability to successfully suppress the wrong response and activate the response required for the correct reaction, which can be observed in the R-LRP signals. Because in incongruent conditions, participants should inhibit an incorrect response first, then the correct response is activated which more effort is required, and the depressed patients are more likely to have functional impairment. If the reduced LRP amplitudes in MD group simply reflect the dysfunction of the inhibition of the incorrect response, it should be evident under the task conditions that cause the stronger activation of the incorrect response (i.e., under incongruent conditions). However, we observed significant reduced LRP amplitudes in MD group under congruent conditions. This founding suggests that depressed patients had reduced LRP amplitudes even under minimal response competition conditions, illustrating that the depressed patients also had functional impairments in activating the correct response.

*The Simon paradigm we chose was to study top-down response choices/conflicts and the propensity to automatic reactions generated by the initiation of visual movements (65–67), both of which are known to be regulated by dopamine (68). We deduce that the reduced R-LRP amplitudes might be associated with low dopaminergic in MD. The low dopaminergic decreases LRP in the primary motor cortex. Therefore, the conflict of choice of reactions, which are considered to be the basis of the Simon effect, is less obvious. Previous study indicated that Parkinson’s is mainly caused by lesions of the dopamine nigrast striatum pathway, and patients with Parkinson’s disease had no reduction in LRP amplitudes (69). However, a decrease in dopamine in the midbrain limbic pathway associated with the reward cannot be ruled out because of a decrease in LRP in MD.*

*The R-LRP amplitudes correlation with the allocation of exercise resources required performing the response, and those were smaller in MD group than in HC group. Therefore, the R-LRP amplitudes may constitute a biomarker in MD. Namely, the response-related effects in this study were not related to the patient’s perceptual stage and the translation process between perception and response.*

In conclusion, patients with MD present conflict control dysfunction (i.e., abnormal cognitive conflict resolution) at the early response-execution stage, not at perceptual encoding stage, which may be reflected by the reduced R-LRP amplitudes. The abnormal cognitive conflict resolution in activating the correct response might constitute an interesting treatment target. However, using logistic regression prediction models, we found that LRP-R under incongruent was more valuable in predicting major depression.

There are some limitations in the study. Firstly, our outcome is preliminary because of a relatively small sample size. Secondly, the internal consistency of ERPs is important when investigating individual differences and that the RIDE method utilized in the present study was not conducive for calculating internal consistency. Therefore, the psychometrics of these specific ERP components remain unclear. Finally, although ERPs have the advantage in temporal resolution, other techniques with better balanced temporal and spatial resolution, such as magnetoencephalography (MEG) should be employed to further investigating the neural mechanism of the cognitive processing of the abnormal cognitive conflict resolution in MD.

## Data availability statement

The raw data supporting the conclusions of this article will be made available by the authors, without undue reservation.

## Ethics statement

The studies involving human participants were reviewed and approved by the Ethics Review Committee of Affiliated Wuxi Mental Health Center of Nanjing Medical University. The patients/participants provided their written informed consent to participate in this study. Written informed consent was obtained from the individual(s) for the publication of any potentially identifiable images or data included in this article.

## Author contributions

Z-HZ: conceived the study. R-HS, JW, and Z-HZ: data curation, formal analysis, methodology, writing-original draft, and writing-review and editing; S-YJ, C-GJ, J-ZZ, X-ZG, and JW: data curation and methodology. All authors contributed to the article and approved the submitted version.
